# The association of circulating sclerostin level with markers of bone metabolism in patients with thyroid dysfunction

**DOI:** 10.5937/jomb0-24943

**Published:** 2020-10-02

**Authors:** Olgica Mihaljević, Snežana Živančević-Simonović, Aleksandra Lučić-Tomić, Irena Živković, Rajna Minić, Ljiljana Mijatović-Teodorović, Zorica Jovanović, Marija Anđelković, Marijana Stanojević-Pirković

**Affiliations:** 1 University of Kragujevac, Faculty of Medical Sciences, Department of Pathophysiology, Kragujevac; 2 University of Kragujevac, Faculty of Medical Sciences, Department of Internal Medicine, Kragujevac; 3 Institute of Virology, Vaccines and Sera, Torlak, Department of Scientific Research, Belgrade; 4 University of Kragujevac, Faculty of Medical Sciences, Department of Nuclear Medicine, Kragujevac; 5 University of Kragujevac, Faculty of Medical Sciences, Department of Biochemistry, Kragujevac

**Keywords:** beta-cross-laps, bone metabolism, osteocalcin, sclerostin, thyroid dysfunction, tireoidna disfunkcija, sklerostin, osteokalcin, koštani metabolizam, beta-cross-laps

## Abstract

**Background:**

The aim of this study was to compare serum sclerostin concentrations in patients with thyroid dysfunction with euthyroid control subjects and to assess the relationship between sclerostin and markers of bone metabolism (osteocalcin and beta-cross-laps).

**Methods:**

The study included 30 patients with thyroid dysfunction (hypothyroidism, hyperthyroidism and subclinical hyperthyroidism) and ten euthyroid controls. Free thyroxine (FT4) was measured by radioimmunoassay, while thyroid stimulating hormone (TSH) concentration was determined immunoradiometrically. We used an ELISA kit to determine the sclerostin level. The electrochemiluminescence method was applied for measuring the bone markers.

**Results:**

Sclerostin levels were significantly lower in hypothyroid patients (p=0.009) and significantly elevated in hyperthyroid patients (p=0.008) compared to control values. Hyperthyroid patients also had higher sclerostin than patients with subclinical hyperthyroidism (p=0.013). Sclerostin concentrations were negatively correlated with TSH levels (r=-0.746, p<0.001), but positively with FT4 (r=0.696, p < 0.001). Moreover, sclerostin was positively associated with osteocalcin (r=0.605, p=0.005) and beta-cross-laps levels (r=0.573, p=0.008) in all thyroid patients.

**Conclusions:**

Serum sclerostin is significantly affected in subjects with thyroid dysfunction. Both sclerostin and thyroid status affect bone homeostasis, which is reflected through the significant correlations with osteocalcin and beta-cross-laps.

## Abstract (sr)

**Uvod:**

Cilj ove studije bio je da uporedimo serumske koncentracije sklerostina kod pacijenata sa disfunkcijom štitaste žlezde u odnosu na eutiroidne kontrolne ispitanike, i da procenimo odnos između sklerostina i markera koštanog metabolizma (osteokalcina i beta-cross-lapsa) u istoj populaciji.

**Metode:**

Studijom je obuhvaćeno 30 pacijenata sa disfunkcijom štitaste žlezde (hipotireozom, hipertireozom i supkliničkom hipertireozom) i 10 eutiroidnih kontrola. Slobodni tiroksin (FT4) meren je radioimunološkom metodom, dok je koncentracija tireostimulišućeg hormona (TSH) određivana imunoradiometrijski. Za merenje nivoa sklerostina koristili smo ELISA-test. Metoda elektrohemiluminiscencije primenjena je za merenje koncentracije koštanih markera.

**Rezultati:**

Nivo sklerostina bio je značajno niži kod bolesnika sa hipotireozom (p = 0,009) odnosno značajno viši kod bolesnika sa hipertireozom (p = 0,008) u poređenju sa vrednostima kod eutiroidnih kontrolnih ispitanika. Pacijenti sa hipertireozom su takođe imali statistički značajno viši nivo sklerostina u odnosu na bolesnike sa supkliničkom hipertireozom (p = 0,013). Pokazana je negativna korelacija koncentracije sklerostina i TSH (r = -0,746, p < 0,001), odnosno pozitivna korelacija sa FT4 (r = 0,696, p < 0,001) kod pacijenata sa tireoidnom disfunkcijom. Štaviše, sklerostin pozitivno korelira sa koncentracijom osteokalcina (r = 0,605, p = 0,005) i beta-cross-lapsa (r = 0,573, p = 0,008) kod ovih pacijenata.

**Zaključak:**

Serumski nivoi sklerostina izmenjeni su kod osoba sa disfunkcijom štitaste žlezde. Sklerostin zajedno sa tireoidnim statusom utiče na koštani metabolizam, što se ogleda u njegovoj značajnoj korelaciji sa osteokalcinom i beta-cross-lapsom.

## Introduction

Bone homeostasis is based on the balance between two coupled processes: bone resorption and bone formation, so any removed damaged bone must be replaced by an equal amount of healthy tissue [1]. There are many influences involved in bone homeostasis, such as genetic factors, growth factors, mechanical load, hormonal and metabolic status.

Thyroid hormones are known to play a key role in the regulation of metabolism and development of different organs, including bones. They are necessary for the maintenance of bone structure and strength and the achievement of peak bone mass [2]. Thyroid hormones have been shown to change the duration of the bone remodelling cycle and to alter relations between bone formation and bone resorption [3]. During bone growth, thyroid hormones have mainly anabolic effects, while they predominantly exert catabolic action in adults [4].

Thyroid-stimulating hormone (TSH) can also influence skeletal development, as the expression of TSH receptors has been demonstrated in both osteoblasts and osteoclasts [5] [6]. It may have a direct effect on bone tissue by inhibition of osteoclastogenesis [7]. Certainly bone homeostasis is influenced by the balanced actions of thyroid hormones and TSH [5].

Sclerostin is an osteocyte-derived protein, encoded by the SOST gene. This regulator of skeletal metabolism acts as an inhibitor of bone formation [8] by stimulating apoptosis of osteocytes and osteoblasts [9]. Sclerostin also reduces bone mineral content and cortical thickness, and thus makes bones less resistant [10]. Previous studies indicated that serum sclerostin is negatively correlated with parathormone and cortisol but positively correlated with calcitonin [11] [12] [13]. However, published data on the relationship between sclerostin and thyroid hormones are scarce and mostly relate to findings obtained in animal studies [14] [15].

In humans, sclerostin has been analyzed in hyperthyroid patients before and during antithyroid therapy [10] [16]. The cross-sectional study of Engler et al. [17] established significant correlations between thyroid hormone excess and faster bone turnover, observed through an increase of bone degradation markers in overt hyperthyroidism. Regarding patients with subclinical hyperthyroidism, various studies have indicated either normal or elevated bone resorption markers [18] [19].

Likewise, available results about sclerostin and markers of bone metabolism also diverge. Some authors demonstrated that sclerostin correlates negatively with both markers of bone formation and bone resorption, while others affirmed a positive correlation only with bone resorption markers [20] [21] [22].

This study aimed to compare serum concentrations of sclerostin in patients with abnormal thyroid function (hypothyroidism, hyperthyroidism and subclinical hyperthyroidism) with values for healthy euthyroid individuals. Possible mutual relationships of serum sclerostin levels and thyroid status, and both sclerostin and thyroid status with bone metabolism markers (osteocalcin and β-cross-laps) were analyzed in all study subjects.

## Materials and Methods

### Study population

The study population included 30 patients with thyroid dysfunction: 23 (79.3%) females and 7 (20.7%) males of mean age 48.31±18.23 yrs. Patients with thyroid gland disorders were divided into three groups: 10 patients with hypothyroidism due to Hashimoto's thyroiditis (HT), 10 patients with hyperthyroidism due to Graves' disease (GD) and 10 patients with subclinical hyperthyroidism due to thyroxine suppressive therapy (differentiated thyroid carcinoma, DTC). Hashimoto's thyroiditis and Graves' disease were diagnosed based on clinical pre sentation, high levels of antithyroid antibodies (561.94± 1022.1 U/mL for TgAbs and 5523±4070 U/mL for TPOAbs in HT and 18.47±18.14 U/mL for TSHRAbs in GD) and TSH concentrations. DTC was diagnosed histologically, according to the main principles of the current WHO classification of thyroid tumours [23]. All DTC patients included in this study had undergone total thyroidectomy and were then treated with fixed nominal activities of sodium (131-I) iodide administered orally according to the EANM guidelines [24]. In all DTC patients, subsequent whole-body scintigraphy detected no regional or distant metastases.

The control group comprised 10 healthy subjects, 9 (90%) females and 1 (10%) male of mean age 46.5±10.23 yrs. All control subjects were evaluated for thyroid function and absence of thyroid antibodies.

None of the study participants had acute infections or other conditions that could affect the tested parameters. After venipuncture, blood samples (5 mL) from patients and control subjects were collected in tubes (Vacutainer) without anticoagulants. Approximately 30 min after venipuncture, serum samples were obtained by centrifugation at 2000 rpm for 15 min and frozen at -20 °C until required for biochemical analysis.

The study was planned according to ethical guidelines following the Declaration of Helsinki. The institutional review committee of Clinical Center Kragujevac approved our study protocol according to local biomedical research regulations. All patients and control subjects gave informed consent prior to enrolment in the investigation.

### Measurement of free thyroxine (fT4) and thyroid-stimulating hormone (TSH)

Free thyroxine (fT4) concentration was measured by radioimmunoassay (RIA, OCFD03-FT4, Cis-Biointernational, France), with a reference range of 7-18 pg/mL. Thyroid-stimulating hormone (TSH) concentration was determined immunoradiometrically (IRMA TSH, INEP, Zemun, Serbia), with a reference range of 0.3-5.5 mU/L. All measurements were made on a Wallac Wizard 1470 Automatic gamma counter (PerkinElmer Life Sciences, WallacOy, 2005, Finland).

### Measurement of serum sclerostin

Serum sclerostin was determined by quantitative sandwich enzyme immunoassay (DSST00 Human SOST Immunoassay, R&D Systems, USA). A monoclonal antibody specific for human sclerostin was precoated onto a microplate. Aliquots of standards and samples were pipetted into the wells for any sclerostin present to be bound by the immobilized antibody. After washing away all unbound substances, an enzyme-linked polyclonal antibody specific for human sclerostin was added to the wells. Following a wash to remove any unbound antibody-enzyme reagent, substrate solution was added for colour to develop in proportion to the amount of sclerostin bound in the initial step. After stopping further development, the colour intensity was measured at 540 nm. Intraassay and interassay coefficients of variance (CV) were 2% and 9.5%, respectively.

### Determination of serum osteocalcin and b-crosslaps

Serum osteocalcin and b-cros-laps were measured by electrochemiluminescence on a *Cobas e 411* analyzer (*Roche Diagnostics*). The reference range for osteocalcin and β-cross-laps were 11-43 ng/mL and 0.010-5.940 ng/mL, respectively.

### Statistical analysis

All values were expressed as mean ± standard deviation (SD). The commercial SPSS version 13.0 for Windows was used for statistical analysis. The significance of differences in the determined parameters between various groups was analyzed by the Independent samples t-test or Mann-Whitney U-test (depending on the distribution). Association between variables was evaluated by the bivariate correlation test and Pearson/Spearman coefficient. Chi-square tests were used to evaluate categorical data. Probability (p) values less than 0.05 were considered to be statistically significant.

## Results

We determined sclerostin concentrations in sera samples from groups of patients with thyroid dysfunction (hypothyroidism, hyperthyroidism and subclinical hyperthyroidism) and compared them with each other and with those for control subjects. Also, we analyzed the relationships of circulating serum sclerostin levels with osteocalcin and β-cross-laps concentrations. There were no significant differences in age or gender distribution among the patients subgroups and control group (hypothyroid group: c^2^ =18.0, p=0.324 for age and c^2^ =1.25, p=0.264 for gender; hyperthyroid group: c^2^ =12.98, p=0.449 for age and c^2^ =1.25, p=0.264 for gender; sub-hyperthyroid group: c^2^ =15.33, p=0.356 for age and c^2^ =1.05, p=0.305 for gender).

**Figure 1:**
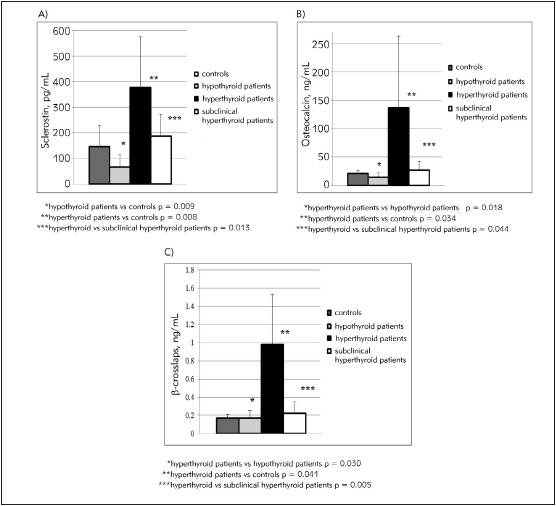
Differences in serum sclerostin (A), osteocalcin (B) and beta-cross-laps (C) concentrations between thyroid patients and control subjects.

Peripheral serum concentrations of sclerostin, free thyroxine and thyroid-stimulating hormone in our study groups are given in Table 1, mean serum sclerostin, osteocalcin and β-cross-laps concentrations are shown in Figure 1 (A, B, C). There were significant differences in the levels of serum sclerostin (Kruskal-Wallis test, p<0.001), osteocalcin (One way ANOVA, p=0.014) and beta-cross-laps (One way ANOVA, p<0.001) between the study groups. We found that patients with hypothyroidism had much lower serum sclerostin concentrations than control subjects (66.54±48.13 pg/mL vs 145.91±81.36 pg/mL) (Mann Whitney test, p=0.009). In contrast, concentrations of sclerostin were significantly higher in patients with hyperthyroidism than in control subjects (377.49±197.44 pg/mL vs 145.91±81.36 pg/mL) (Independent samples t-test, p=0.008) and patients with subclinical hyperthyroidism (Mann Whitney test, p=0.013). Hyperthyroid patients had higher concentrations of osteocalcin and beta-crosslaps in relation to patients with hypothyroidism (p=0.018 for osteocalcin; p=0.030 for beta-crosslaps), patients with subclinical hyperthyroidism (p=0.044 for osteocalcin; p=0.005 for beta-crosslaps) and control subjects (p=0.034 for osteocalcin; p=0.041 for beta-cross-laps).

**Table 1:** Concentrations (geometric mean) of sclerostin, fT4 and TSH in the study groups. The values in parentheses represent the 95% confidence interval of geometric mean, and the differences were considered statistically different if *p*-value was < 0.05.

Concentrations	Experimental group			Control group	Sig.
	Hypothyroid group (n=10)	Hyperthyroid group (n=10)	Subhyperthyroid group (n=10)	Physiologically healthy (n=10)	
Sclerostin (pg/mL)	54.52 (33.79–79.44)	317.12 (188.90–476.50)	175.39 (147.28 –226.21)	125.31 (87.36 –177.41)	*p*< 0.05
fT4 (pg/mL)	0.89 (0.450–1.689)	59.54 (36.45–103.97)	16.56 (14.12–20.04)	9.58 (8.65 –10.64)	*p*< 0.05
TSH (mU/L)	93.63 (62.22–136.83)	0.13 (0.11–0.156)	0.24 (0.19–0.30)	1.67 (1.45 –1.89)	*p*< 0.05

**Figure 2:**
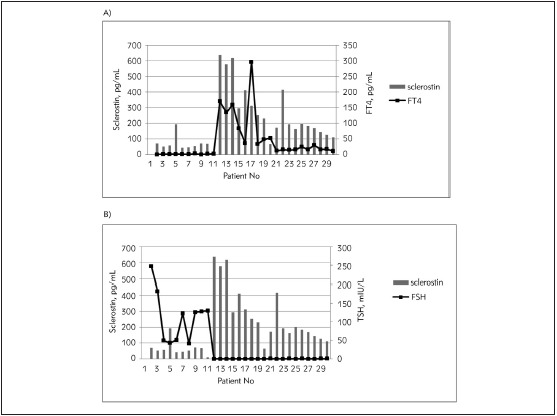
The relationship of sclerostin level with free thyroxine (A) and thyroid-stimulating hormone (B) in subjects with thyroid dysfunctions.

Analysis of the relationship between sclerostin and FT4 showed a strong positive correlation of sclerostin concentrations and free thyroxine in the subjects with thyroid dysfunctions (Bivariate correlation test, Spearman r=0.696, p<0.001) (Figure 2A). On the contrary, serum sclerostin was negatively correlated with TSH in the same participants (Bivariate correlation test, Spearman r=-0.746, p<0.001) (Figure 2B). In the group of control subjects, the correlations of sclerostin and parameters of thyroid status tended in the same directions as for the patients, i.e. positive with FT4 and negative with TSH, but did not reach the level of statistical significance (r_TSH_ =-0.490, p_TSH_ =0.150; r_FT4_ =0.382, p_FT4_ =0.386).

When considering the relationship of bone turnover markers and thyroid status, statistical analysis showed that osteocalcin and beta-cross-laps positively correlated with FT4 (Spearman r = 0.641, p = 0.003 for osteocalcin; Spearman r=0.522, p=0.022 for beta-cross-laps) but negatively correlated with TSH (Spearman r =-0.735, p<0.001 for osteocalcin; Spearman r =-0.612, p=0.004 for beta-cross-laps) in the patients with altered thyroid function.

Finally, sclerostin concentrations were positively associated with both markers of bone metabolism (osteocalcin and beta-cross-laps) in the patients with abnormal thyroid function (Spearman r=0.605, p=0.005 for osteocalcin; Spearman r=0.573, p=0.008 for beta-cross-laps). The relationship between serum sclerostin and osteocalcin (Spearman r=0.131, p=0.779) as well as sclerostin and betacross-laps (Spearman r=0.337, p=0.459) in control subjects was not statistically significant probably due to the small number of subjects (n=10) in this group.

## Discussion

The aim of our study was to evaluate sclerostin concentrations in patients with abnormal thyroid functions (hypothyroidism, hyperthyroidism and subclinical hyperthyroidism) and healthy euthyroid subjects, as well as to analyze the relationship between sclerostin concentrations and markers of bone metabolism (osteocalcin and beta-cross-laps).

Thyroid hormones are considered important regulators of bone metabolism, so any deficiency or excess may result in disturbance of bone turnover. Current knowledge about the action of thyroid hormones on the skeleton has mainly been obtained from animal models [5]. Since bone remodelling determines skeletal integrity, thyroid hormones might change the rate of bone turnover (leading to an acceleration in hyperthyroidism or deceleration in hypothyroidism) and bone density [3]. On the other hand, it is accepted that TSH can suppress osteoclast formation and stimulate osteoblast differentiation [6]. Abe and coworkers [5] hypothesized that TSH, rather than thyroxine and triiodothyronine, is a direct negative regulator of skeletal development. It seems that skeleton maintenance is set by the concerted activity of thyroid hormones and TSH.

Sclerostin is a glycoprotein product of the SOST gene expressed mainly in osteocytes [8]. It has a negative effect on bone mineralization because it reduces bone mineral content and has been the subject of many studies. Thus, increased serum sclerostin levels were found in patients with chronic kidney disease [25], diabetes mellitus [26], prostate cancer [27], liver dysfunction [28] and obesity [29]. Its expression is regulated mostly by mechanical load, although many data indicate the great importance of humoral factors [10] [12] [13]. Thus, thyroid hormones were demonstrated to increase the number of sclerostinpositive osteocytes in hyperthyroid mice [14] [15]. Here we attempted to clarify the relationship of sclerostin and thyroid hormones in humans and are the first to show differences in circulating sclerostin concentrations in patients with three different types of thyroid dysfunction (hypothyroidism, hyperthyroidism and subclinical hyperthyroidism).

In earlier studies on sclerostin concentrations in subjects with hyperthyroidism before and/or after medicament therapy, Skowrońska-Jóźwiak et al. [10] and Sarıtekin et al. [16] found no statistically significant correlation with thyroid status. Unlike them, we have compared serum sclerostin concentrations in patients with different abnormalities of thyroid function both between the groups of patients and with healthy euthyroid individuals.

We showed that serum sclerostin concentrations were the highest in the group of hyperthyroid patients, significantly higher than in control subjects. On the contrary, patients with hypothyroidism had lower serum concentrations of sclerostin than the other groups. These results are in line with data obtained by Tsourdi et al. [15] in male mice. It seems that hypothyroidism, although this may not be expected, affects bone homeostasis and reduces skeletal turnover.

Our study also included DTC patients with subclinical hyperthyroidism caused iatrogenically by thyroxine suppressive therapy. Subclinical hyperthyroidism is characterized by circulating TSH levels below the reference range and normal serum concentrations of thyroid hormones. The small difference in sclerostin levels between these patients and control subjects was not statistically significant, although the mean concentration was slightly higher in patients with subclinical hyperthyroidism. These results are in agreement with those of Lee et al. [30], who concluded that TSH suppression in DTC patients has no effect on bone health, so suppressive levothyroxine therapy could be rated safe.

A close positive correlation of sclerostin concentrations with thyroxine and a strong negative correlation with TSH in sera samples from our three groups of patients were observed. The same correlation trend of sclerostin and thyroid status (FT4 and TSH) was seen in control subjects but did not reach statistical significance, probably due to the small number of subjects in the control group.

Analysis of the relationship with bone metabolic markers showed that serum sclerostin was positively associated with osteocalcin and beta-cross-laps levels in patients with thyroid dysfunctions. Moreover, osteocalcin and beta-cross-laps were positively associated with concentrations of FT4 and negatively with TSH in all patients.

Osteocalcin is the most common non-collagenous protein of the bone extracellular matrix. Synthesized by mature osteoblasts [31], it is considered a marker of bone formation. Thus, serum osteocalcin level correlates with osteoblast activity, although fragments can be released into the blood during bone resorption. In contrast, beta-cross-laps is a collagen-degradation product and a marker of bone resorption [32].

In an analysis of osteocalcin concentrations in patients with abnormal thyroid function (hyperthyroidism, hypothyroidism and subacute thyroiditis), Kojima et al. [33] found a positive correlation with thyroxine and triiodothyronine levels, suggesting that osteoblast activity was increased in hyperthyroidism and decreased in hypothyroidism. Moreover, thyroid suppressive therapy significantly reduced osteocalcin concentrations in subjects with hyperthyroidism [34]. Our results correspond with these findings, but we have also demonstrated that both osteocalcin and beta-cross-laps are associated with thyroid status in patients with different thyroid dysfunctions. Furthermore, we have examined the relationship of sclerostin with other bone metabolism markers, which was not the subject of previous research. Earlier results about the association of sclerostin and markers of bone metabolism mostly concerned a healthy elderly population or immobilized subjects. Thus some authors demonstrated that sclerostin correlates negatively with markers of bone formation and bone resorption, while others affirmed a positive correlation only with bone resorption markers [20] [21] [22]. Unlike Sarıtekin et al. [16], our examination indicated positive relationships of serum sclerostin with both osteocalcin and beta-cross-laps in patients with abnormal thyroid function. The differences in sclerostin concentration in our study participants reflect changes in the level of osteocalcin and beta-cross-laps and vice versa. As a marker of bone formation, osteocalcin showed the same distribution among our study groups as sclerostin and beta-cross-laps (the highest values in hyperthyroid patients, the lowest in hypothyroid patients). We assume that is a consequence of a compensatory reaction (as increased osteoblast activity) in the case of disturbed bone balance. This has been shown in some other conditions, e.g. in subjects with Wilson's disease and postmenopausal females [35] [36].

### Study limitations

The principal limitation of our study is the relatively small samples size. Secondly, wide confidence intervals of the selected associations indicate a limited statistical strength. Thus, further examinations are needed in order to verify the strength of the observed associations.

In conclusion, serum concentrations of sclerostin are significantly affected in subjects with different abnormal thyroid functions. Thus, patients with hyperthyroidism had the highest level of sclerostin and patients with hypothyroidism the lowest. There were positive relationships between circulating concentrations of sclerostin and markers of bone metabolism, i.e. osteocalcin a marker of bone formation and beta-cross-laps marker of bone resorption in all thyroid patients. Thyroid status affects bone homeostasis which is reflected through its significant association with osteocalcin and beta-cross-laps but even more closely with sclerostin.

*Acknowledgements*. The study was supported bythe Ministry of Education, Science and Technological Development of the Republic of Serbia (Grant Nos. III41010 and ON175069) and the Faculty of Medical Sciences, University of Kragujevac, Serbia (JP 06–12).

## Conflict of interest statement

The authors state that they have no conflicts of interest regarding the publication of this article.

## List of abbreviations

DTC, differentiated thyroid carcinoma; GD, Graves’ disease; HT, Hashimoto’s thyroiditis.
